# Is CRT Optimization Obsolete? A Referral Center’s Experience

**DOI:** 10.31083/j.rcm2502063

**Published:** 2024-02-18

**Authors:** Shmaila Saleem-Talib, Mirjam D. Duineveld, Jurjan C. Schipper, Arnaud D. Hauer, Hemanth Ramanna, Natasja M.S. de Groot, Michael G. Scheffer

**Affiliations:** ^1^Department of Cardiology, Haga Teaching Hospital, 2545 AA The Hague, The Netherlands; ^2^Department of Cardiology, Reinier de Graaf Gasthuis, 2625 AD Delft, The Netherlands; ^3^Department of Cardiology, Erasmus Medical Center, 3015 GD Rotterdam, The Netherlands

**Keywords:** CRT-optimization, cardiac resynchronization therapy, strain rate imaging

## Abstract

**Background:**

Cardiac resynchronization therapy (CRT) is a well-established therapy for patients with heart failure (HF). However, 30% of HF patients do not show any improvement in clinical status after CRT implantation. In this study, we report our echocardiography-based CRT optimization methodology, in daily practice at our CRT referral center.

**Methods:**

We included 350 ambulatory patients, who were referred to our center for optimization after CRT implantation. A protocol-driven echocardiographic approach for adjusting mechanical dyssynchrony, whereby adjusting for ventriculoventricular (VV) delays with strain and atrioventricular (AV) delays with Doppler echocardiography was performed. We defined changes in left ventricular ejection fraction (LVEF) and New York Heart Association (NYHA) classes as outcome variables in the evaluation of the CRT outcomes.

**Results:**

Optimization was obtained in 288 (82%) patients. VV and AV timings were adjusted to 61% and 51%, respectively. In 3%, biventricular pacing was turned off and in 3% left ventricular (LV) only pacing was programmed. The LVEF and NYHA class showed significant improvements in all patients who underwent CRT optimization.

**Conclusions:**

CRT optimization remains valuable in improving LVEF and functional status measured using the NYHA class in all patients receiving CRT devices.

## 1. Introduction

Cardiac resynchronization therapy (CRT) is a well-established therapy for patients with heart failure (HF) with a reduced left ventricular ejection fraction (LVEF) ≤35% and interventricular conduction delay with a broad QRS ≥130 ms [1, 2, 3, 4, 5]. Although guidelines provide criteria for selecting patients who will benefit the most from this therapy [4, 5, 6, 7], still one third (30%) of patients do not respond to CRT [4, 8, 9]. This leaves us questioning the optimal management of patients who already have a CRT and whether optimizing CRT makes a difference [10, 11].

This article reports our experience with a protocol-driven echocardiography-based CRT optimization program in all patients who were referred to our center for CRT optimization.

## 2. Methods

This is a retrospective observational single-center study that evaluates the outcomes of our CRT optimization program, which started in 2012. All consecutive ambulatory patients with CRT devices who were referred to our center for optimization were included. The patients were seen at baseline by a multidisciplinary team consisting of an HF nurse, an echocardiographer, a device technician, and a cardiologist specializing in device implantations. A total of 350 patients were included, 20% of whom had undergone cardiac resynchronization therapy with a pacemaker (CRT-P) and 80% who had cardiac resynchronization therapy with an implantable cardioverter defibrillator (CRT-D). Selection criteria for receiving a CRT were implemented according to the ESC or ACC/AHA/HRS guidelines [6, 7].

Preimplantation LVEF and New York Heart Association (NYHA) class, were provided by the referring cardiologist. All included patients were referred to us from other centers after CRT implantation and with the optimization of HF drugs. Patients were treated with angiotensin-converting enzyme (ACE) inhibitors and beta blockers to the maximum tolerated doses.

During the first clinical visit, each patient was examined by a multidisciplinary team. Antiarrhythmic drugs were introduced to reduce the burden of premature ventricular contractions (PVC) or to undergo treatment for the upper rate behavior to achieve >99% biventricular pacing.

We also performed a chest X-ray to confirm the lead positions, in particular, to confirm the position of the left ventricle (LV) lead.

The NYHA class was assessed by the HF nurse and the LVEF was measured by the echocardiographer using the biplane Simpson method, recommended by the European Association of Cardiovascular Imaging and the American Society of Echocardiography [12].

Six months after optimization, patients were re-evaluated by the multidisciplinary team. Previous studies have shown that the benefit of CRT was most often seen within the first 3–6 months after implantation [1, 8, 13].

At baseline, patients were defined by the referring cardiologist as being either responders (R) or non-responders (NR) to CRT. This was based on both the improvement in the NYHA class and an increase in LVEF. If the NYHA class improved in a patient by at least one class and the LVEF improved by at least 15%, compared to the values measured before CRT implantation, they were considered an R to CRT. If only the NYHA class improved, with no improvement observed in the LVEF during echocardiography, using the biplane Simpson method, then these patients would be considered NR, as demonstrated in previous studies [9, 11, 14]. Both R and NR patients were referred to our center for CRT optimization. Patients denoted an R were also referred to us so that the maximum benefit of CRT could be achieved.

### 2.1 Device Interrogation

The device was routinely interrogated at the first visit, including the percentage of biventricular pacing. The goal was to achieve >99% biventricular pacing by addressing the cause of suboptimal biventricular pacing due to fusion pacing, ventricular and atrial arrhythmias, and upper-rate behavior.

### 2.2 Echocardiographic Optimization

**Fig. 1.**
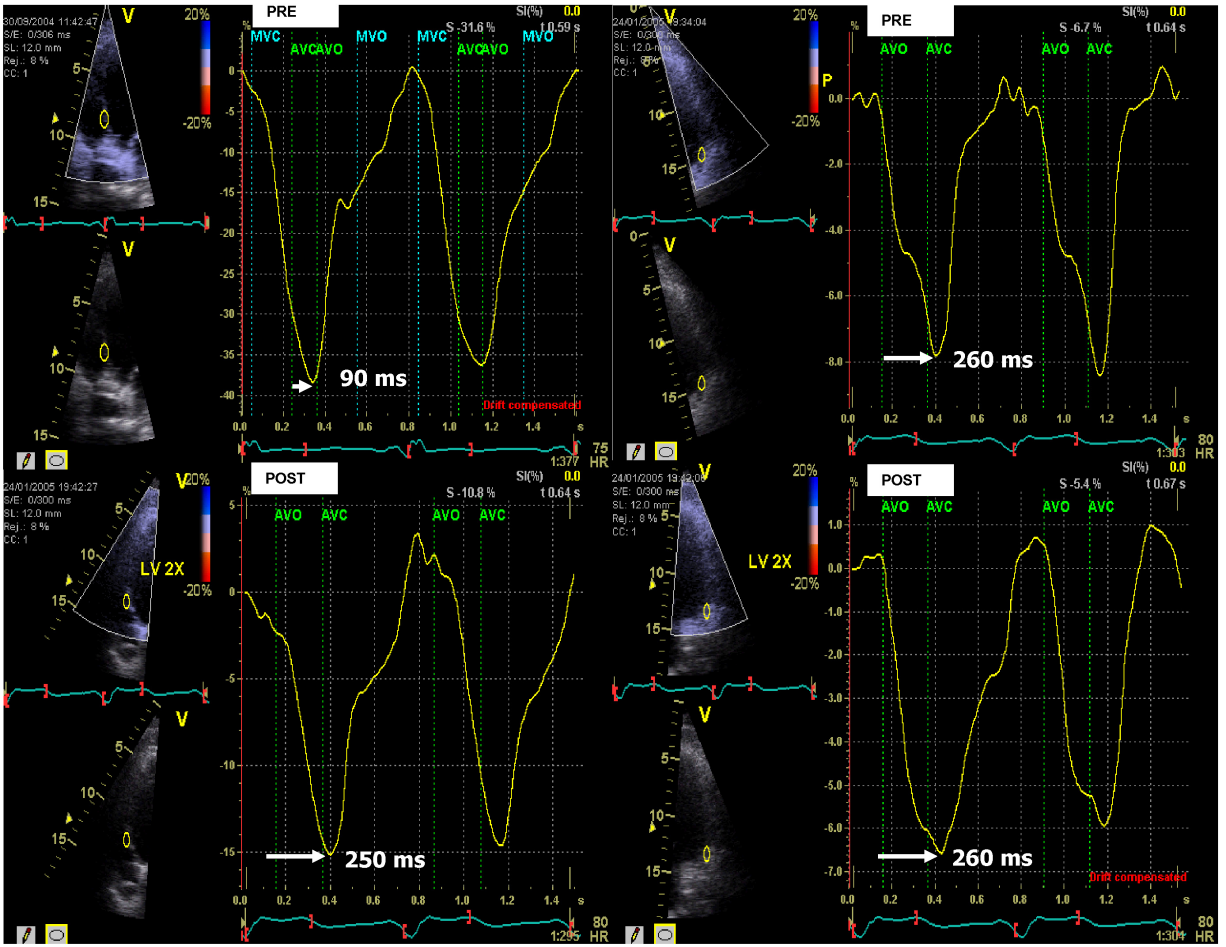
**Measuring left ventricular (LV) dyssynchrony with peak longitudinal strain derived from strain rate imaging (SRI)**. The method used to measure dyssynchrony with SRI. Peak longitudinal strain curves from the mid-septum (left panel) and mid-lateral wall (right panel) in a patient undergoing mechanical delay pre-cardiac resynchronization therapy (CRT) optimization of 260 ms – 90 ms = 170 ms, measured from the opening of the aortic valve to the maximal strain of the mid-septum and mid-lateral wall in the left ventricle. Mechanical dyssynchrony is exhibited. Post-CRT optimization of the mechanical delay between the mid-septum and the mid-lateral walls was reduced to 260 ms – 250 ms = 10 ms. AVC, aortic valve closed; AVO, aortic valve open; MVC, mitral valve closed; MVO, mitral valve open.

A conventional echocardiographic examination was conducted following device interrogation to evaluate the LVEF, valvular dysfunction, and mechanical dyssynchrony. The LVEF was measured using the biplane Simpson method at baseline echocardiography and again 6 months after optimization [12]. To identify the initial mechanical dyssynchrony, the device was programmed to atrial and ventricular sensing if possible. Mechanical dyssynchrony was assessed using strain rate imaging (SRI), previously derived from tissue Doppler imaging (TDI). This method was performed using an apical four-chamber view. The image sector width was chosen to be as narrow as possible with an angle of 10 to 20 degrees in the mid-septum and mid-lateral wall separately to achieve the highest acquisition frame rates (200–250 fps) and avoid aliasing. Regional strain rates were estimated from the spatial gradient in the myocardial velocity profile over a user-defined sample volume with a computational sample of 10 mm. The regional strain rate profiles were integrated over time to obtain the natural systolic strain profile [15]. Aortic and mitral flow measurements were performed using pulsed wave Doppler (PD) to identify opening and closure timings of the aortic and mitral valves. These timings would provide the exact measurement from the onset of the aortic opening to the maximal peak strain from the mid-septal and the mid-lateral walls. The intraventricular mechanical delay was measured using the SRI, based on the difference between these two measurements (Fig. 1).

Optimization of the ventriculoventricular (VV) timing was guided by the natural systolic strain profile derived from SRI (Fig. 1). Optimal VV timing was considered when the septal and lateral peak strains showed the smallest time difference at the closure of the aortic valve [16]. Optimization of the atrioventricular (AV) delay was performed using the iterative method based on the E/A ratio, measured by PD. Since considerable hemodynamic effects can occur when the AV delay is either too short or too long, this method allowed an optimal AV delay to be chosen [14, 17, 18].

Echocardiographic examinations were performed using the General Electric Vived E9 or the System Seven (GE Vinghmed Ultrasound, Horten, Norway) with a 2.5 and 3.5 MHz multiphase transducer. Two-dimensional and M-mode echocardiographic images were obtained according to the guidelines of the European Association of Cardiovascular Imaging and the American Society of Echocardiography [19].

### 2.3 Clinical Assessment

An HF practitioner evaluated each NYHA classification before and after optimization. A response to CRT was defined as an improvement in NYHA classification by one or more classes and an increase in LVEF of ≥15%.

### 2.4 Statistical Analysis

Continuous variables are expressed as mean ± standard deviation (SD) when normally distributed, otherwise, they are expressed as the median and interquartile range (IQR). Ordinal variables are expressed as counts with IQR and percentages. To detect differences in the NYHA class and LVEF, we used the sign test and the Wilcoxon signed-rank test. Two-sided *p*-values smaller than 0.05 were considered statistically significant. The chi-square test was used for the categorical variables, while ANOVA (Analysis of Variance) was performed to analyze the differences between more than two groups. For all analyses, R software (version 3.2.0, R Foundation for Statistical Computing, Vienna, Austria) was used.

## 3. Results

Patient characteristics are summarized in Table 1. We included 350 patients, of which 229 patients (65%) were male with a mean age of 73 years, while 121 (35%) were female with a mean age of 70 years. The baseline LVEF values were missing in six patients, meaning they were included in the NYHA class improvement analyses but excluded from the LVEF improvement analysis.

**Table 1.** **Baseline characteristics**.

Baseline Characteristics		N = 350	%
Gender			
	M	229	(65)
	F	121	(35)
Age (yrs)			
	mean (SD)	72 (10)	
		N	SD
	M	73	(10)
	F	70	(10)
NYHA classification		N = 350	%
	I	0	0
	II	74	(21)
	III	250	(71)
	IV	26	(7)
NICMP		N = 140	%
	M	60	(43)
	F	80	(57)
Age (yrs)			
	mean (SD)	69 (12)	
ICMP		N = 210	%
	M	169	(80)
	F	41	(20)
Age (yrs)			
	mean (SD)	74 (9)	
LVEF		N = 344	
	median (IQR)		%
	10–20%	65	(19)
	20–30%	127	(37)
	30–40%	123	(36)
	40–50%	29	(8)

Abbreviations: N, number; yrs, age in years; M, male; F, female; SD, standard deviation; IQR, interquartile range; NYHA, New York Heart Association classification; NICMP, non-ischemic cardiomyopathy; ICMP, ischemic cardiomyopathy; LVEF, left ventricular ejection fraction.

Our population consisted of 184 (53%) R and 160 (47%) NR at baseline.

Underlying cardiac diseases in patients included: 140 patients (40%) with non-ischemic cardiomyopathy (NICMP) and 210 patients (60%) with ischemic cardiomyopathy (ICMP). In the ICMP subgroup, 169 patients were male (80%) and 41 were female (20%). In the NICMP subgroup, 60 patients were male (43%) and 80 patients were female (57%). Patients in the ICMP subgroup were older than the patients in the NICMP subgroup (*p* < 0.01). Twenty-nine patients had an LVEF between 40–50% before implantation; these are patients that received a CRT due to secondary prevention after cardiac arrest and a high percentage of expected ventricular pacing due to a second or third-degree AV-block. Patients were referred to our center after optimizing the use of the HF drugs. During the optimization process, we optimized the use of antiarrhythmic drugs to reduce the PVC burden and to treat upper-rate behavior to achieve biventricular pacing of >99%. Unfortunately, optimization failed in 15 patients due to the presence of tachyarrhythmias. At baseline, 147 patients (42%) received biventricular pacing of less than 99%.

In our study population, the prevalence of medication usage was as follows: amiodarone (25%), beta blockers (57%), statins (49%), ACE inhibitors (71%), angiotensin receptor blockers (15%), diuretics (78%), and digoxin (27%). The mean biventricular paced QRS width before and after optimization was 165 ms (SD 28 ms) and 153 ms (SD 24 ms), respectively, *p* < 0.001.

### 3.1 Optimizing CRT Settings

We also performed a chest X-ray to confirm the position of the leads, in particular to confirm the position of the LV lead.

The LV lead placement was posterolateral in 74% of the participants, lateral in 24%, and anterolateral in 2%. In patients with an anterolateral positioned LV lead, VV optimization could not be achieved, meaning only AV was optimized.

During the follow-up at 6 months, the mean of the sensed AV delay was programmed to 103 ms (SD 22 ms) and the mean of the paced AV delay to 128 ms (SD 25 ms). Furthermore, the mean of the VV delay was programmed to 21 ms (SD 19 ms).

**Fig. 2.**
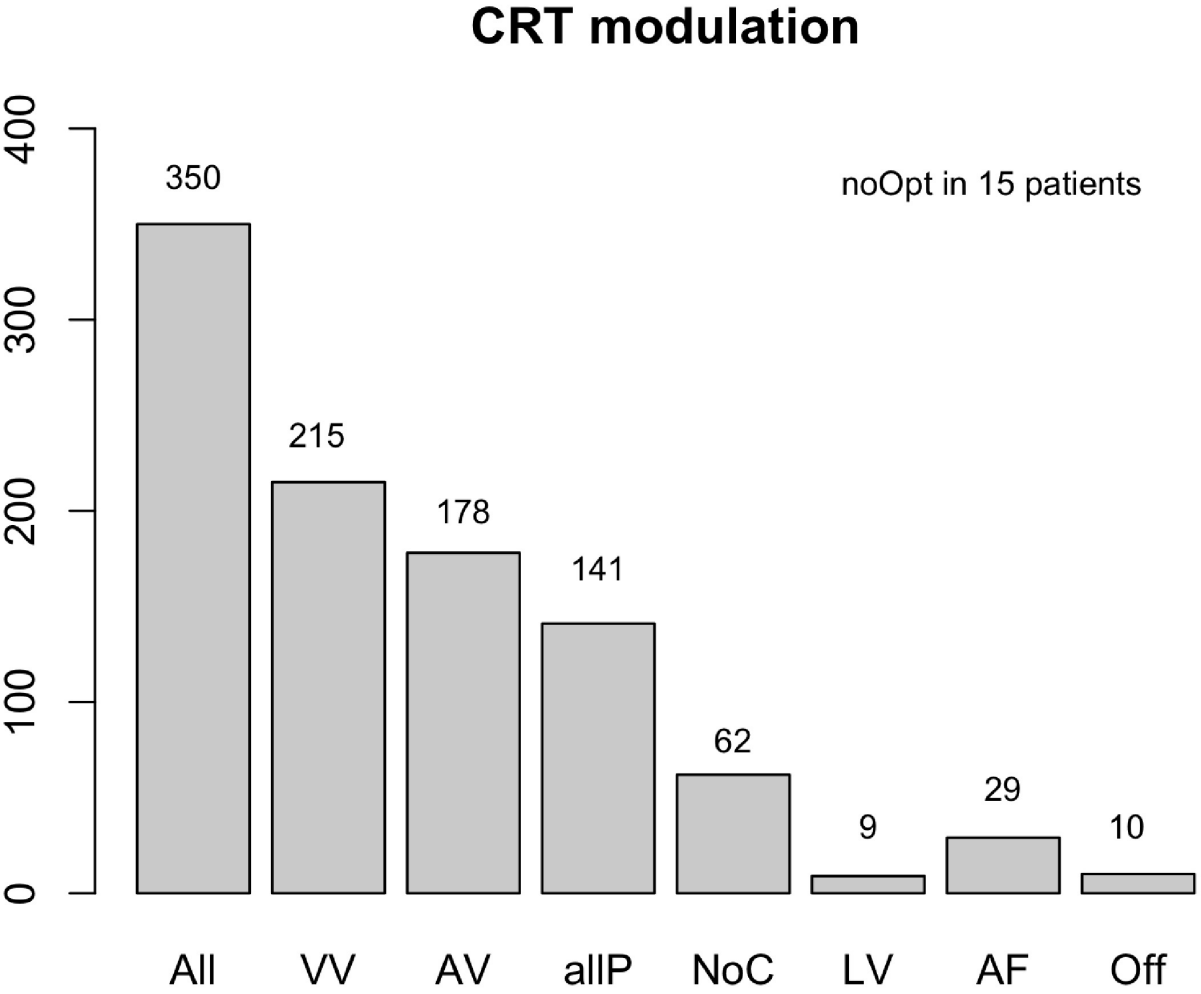
**CRT modulation**. Abbreviations: N, number (n = 350); VV, VV interval programming; AV, AV interval modification; allP, VV, and AV combined; NoC, no changes were necessary; LV, LV only pacing; AF, VV interval adaptation in patients with atrial fibrillation; Off, pacing mode switched off; noOpt, no changes could be made. Suboptimal VV intervals were adjusted in 215 patients, while the AV settings were modified in 178 patients according to the iterative method. Both VV and AV interval (allP) optimizations were performed in 141 patients, while no changes were necessary (NoC) in 62 patients. Device settings were already optimal at baseline. CRT was programmed in 9 patients to LV only. In 29 patients, VV programming only could be performed due to underlying atrial fibrillation (AF). The CRT was turned off in 10 patients because they did not have dyssynchrony (Off) at baseline. Furthermore, optimization failed (noOpt) in 15 patients due to arrhythmias. VV, ventriculoventricular; AV, atrioventricular; LV, left ventricle; CRT, cardiac resynchronization therapy.

In 82% of the CRT patients (N = 288), suboptimal VV or AV settings were found. Optimization of the VV interval was achieved in most patients 215 (61%), with a considerable amount of 178 (51%) also exhibiting adaptations in AV timings. Optimization of both the VV and AV intervals (aIIP) was performed in 141 (40%) patients (Fig. 2).

### 3.2 LVEF and NYHA Class After Optimization

**Fig. 3.**
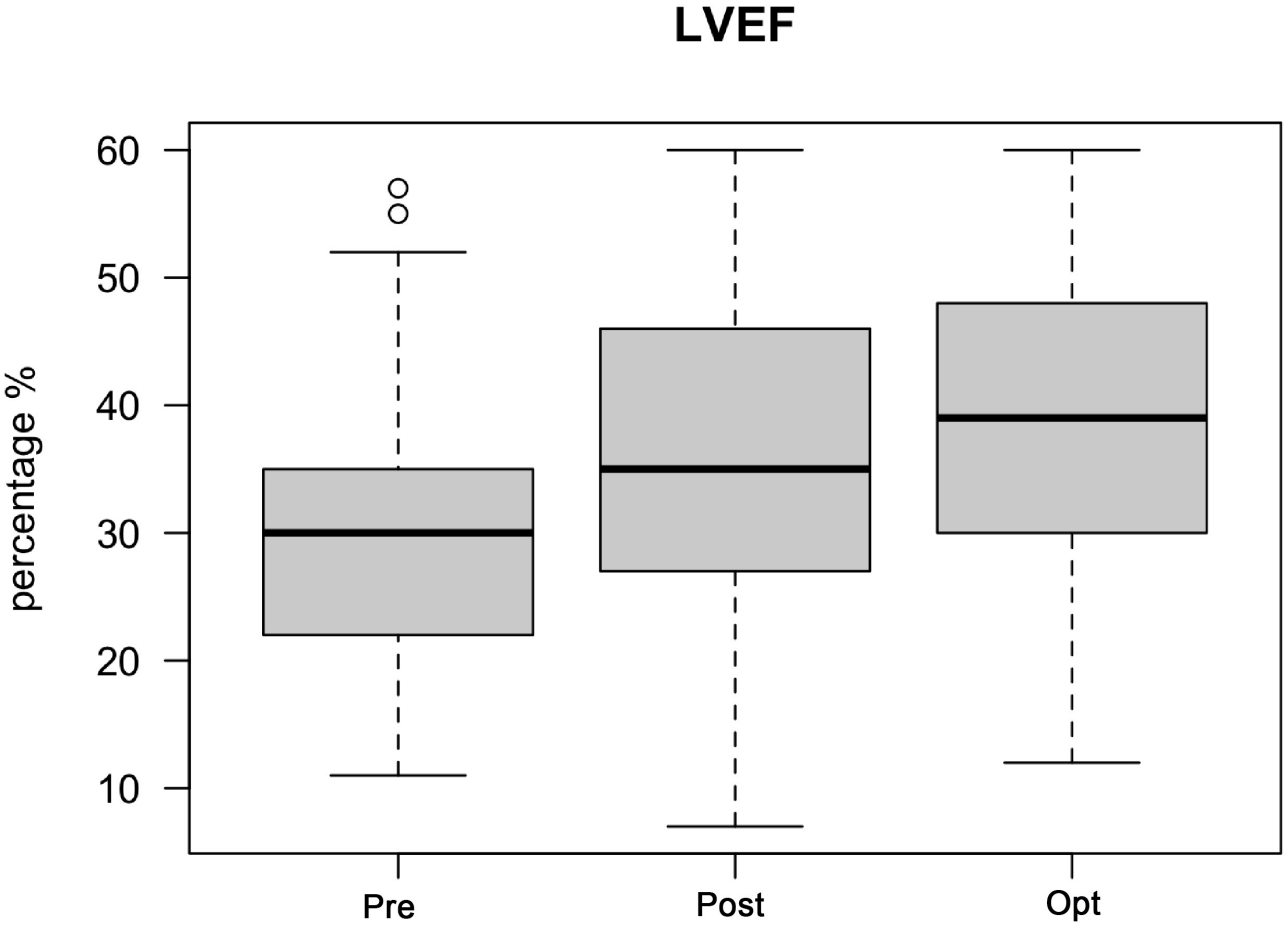
**LVEF improvement after CRT optimization**. Abbreviations: LVEF, left ventricular ejection fraction; CRT, cardiac resynchronization therapy; Pre, before CRT implantation; Post, after CRT implantation but before CRT optimization; Opt, after CRT optimization. The median LVEF prior to CRT implantation was 30%. After implantation, there was a significant improvement in median LVEF to 35%. After CRT optimization, the median LVEF further improved to 39% (*p* < 0.04). There were 344 patients with LVEF measurements available.

The median LVEF prior to CRT implantation was 30%. After implantation, there was a significant improvement in the median LVEF to 35%. After CRT optimization, the median LVEF further improved to 39% (*p* < 0.04). LVEF measurements were available in 344 patients, while the LVEF improved after CRT optimization in 216 patients (63%). The LVEF deteriorated in 6 patients (2%), whereas the LVEF did not change in 122 patients (35%) after optimization (Fig. 3).

**Fig. 4.**
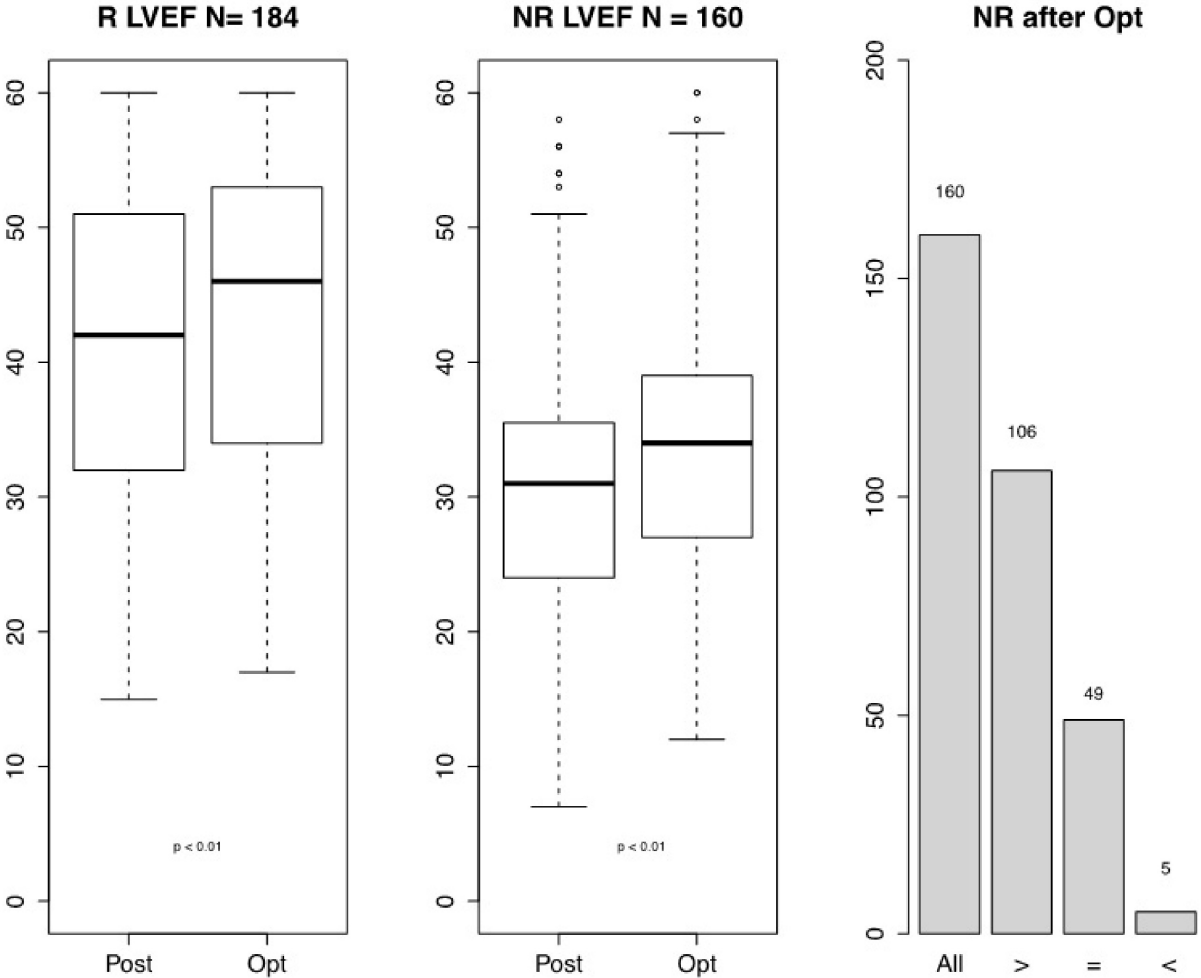
**LVEF improvement after CRT optimization in responders and non-responders**. Abbreviation: N, number; R LVEF, responders left ventricular ejection fraction; NR LVEF, non-responders left ventricular ejection fraction; NR after Opt, number of non-responders after optimization; All, all non-responders; >, increase in LVEF; =, no difference in LVEF; <, decrease in LVEF; Post, post CRT implantation but before CRT optimization; Opt, after CRT optimization; CRT, cardiac resynchronization therapy. Based on the selection criteria for R and NR, there were 184 R and 160 NR at baseline. LVEF showed significant improvements before and after optimization in both the R and NR groups.

Differences between responders and non-responders: Based on the selection criteria for the R and NR, 184 patients (53%) were designated at baseline as R, with 160 (47%) NR. LVEF showed significant improvements before and after optimization in both the R and NR groups. In the NR group, 106 patients exhibited an improvement in LVEF, while the LVEF remained stable in 49 patients, whereas the LVEF declined in 5 patients after optimization (Fig. 4).

## 4. Discussion

This study shows that we can manage patients who have previously received a CRT device and are referred to our referral center for CRT optimization. Prior to achieving CRT optimization, it was important that patients received optimal medical therapy with the optimization of HF drugs and that the position of the LV lead was known. Then, CRT optimization was performed through two simple steps.

The first step was device interrogation, which was performed to achieve >99% biventricular pacing, while the second step was conducted to address the mechanical dyssynchrony as measured by echocardiography. We showed that optimizing CRT devices provides additional value in further improving the LVEF and NYHA classes in all patients.

Currently, there is no consensus on how to optimize the CRT settings or how to measure LV dyssynchrony. Electrical dyssynchrony is not equivalent to mechanical dyssynchrony and a reliable non-invasive parameter to detect mechanical dyssynchrony in CRT patients is lacking [20, 21].

The PROSPECT trial demonstrated that, of the 12 tested echocardiographic parameters, none had the diagnostic power to predict the responsiveness to CRT [22]. We showed that peak longitudinal strain derived from SRI was a superior measure for LV dyssynchrony and that peak longitudinal strain delay between the mid-septum and mid-lateral walls was superior for measuring mechanical dyssynchrony [16]. We believe this method improved our accuracy in measuring LV dyssynchrony, meaning it can lead to improved VV synchronization after CRT optimization.

We believe that the biggest advantage of echocardiography-based optimization is the adjustment of AV delays based on the iterative method with the E/A ratio measured by echocardiographic examination, during which the effects of AV delays that are both too short and too long can be measured and adjusted according to the best E/A ratio. Moreover, the assessment of LV dyssynchrony with peak longitudinal strain delay measurements and the adjustment of VV delays accordingly is very accurate. We did not compare echocardiography-based optimization with QRS-based optimization since electrical dyssynchrony is not equivalent to mechanical dyssynchrony and does not accurately predict ventricular dyssynchrony, as shown by Bleeker *et al*. [21]. However, we acknowledge that the reduction in QRS width occurred in this cohort following VV optimization.

In total, 82% of patients with a CRT device received CRT optimization after implantation. We found an improvement in the LVEF and NYHA classes after CRT implantation, which has also been demonstrated in earlier studies [1, 2, 3]. After further optimization, patients experienced improvements in both the clinical status and the mean LVEF, which improved from 35% to 39%, *p* < 0.04 (Table 2). Although CRT is a well-established treatment for HF patients with reduced ejection fraction (HFrEF), one-third (30%) of HFrEF patients still do not respond to this therapy [4, 8, 9]. In this study, a population of 160 patients (47%) were denoted as NR, which is more than was expected based on the previous literature. This difference may be because our clinic is a referral center, which means physicians are more likely to refer NR patients to our clinic for optimization.

**Table 2.** **Differences between responders and non-responders**.

Variables		Before	After	*p*-value
LVEF all (N = 344)				
	median (IQR)	35 (18)	39 (19)	<0.04
LVEF R (N = 184)				
		42 (19)	46 (19)	<0.02
LVEF NR (N = 160)				
		31 (11)	34 (12)	<0.05
NYHA all (N = 350)		N	N	<0.02
	I	1	104	
	II	120	198	
	III	223	46	
	IV	6	2	
NYHA R (N = 184)				
	I	0	65	<0.04
	II	72	100	
	III	107	18	
	IV	5	1	
NYHA NR (N = 160)				
	I	0	39	<0.05
	II	49	96	
	III	110	24	
	IV	1	1	

Abbreviations: LVEF, left ventricular ejection fraction; NYHA, New York Heart Association classification; Before, after CRT implantation but before CRT optimization; After, after CRT optimization; all, both responders and non-responders; R, responders; NR, non-responders; N, number of patients; IQR, interquartile range; CRT, cardiac resynchronization therapy.

The VV timing was also investigated in this study. The VV timing required adjustment in 61% of the patients. Inadequate VV timings cause more LV dyssynchrony and lead to a reduced stroke volume [23]. We showed that 51% of the CRT patients had suboptimal programming of the AV timing, which led to inefficient LV filling. Thus, biventricular pacing is an absolute necessity to optimally synchronize the LV to >99% [24]. Therefore, we aimed for >99% biventricular pacing after optimization. We found that 42% of the patients did not meet this requirement due to AF or supraventricular or ventricular ectopy. Our results are in accordance with the results of Mullens *et al*. [14].

We understand that echocardiography-based optimization is not widely implemented due to its time-consuming nature. Recent research has shown that device-based algorithms might simplify CRT optimization and lead to patient-tailored optimization following improvements in electrical synchrony [25].

### Study Limitations

This study describes our CRT optimization program. Since we aimed to optimize all patients with a CRT device, this study did not contain a control group.

We present the advantages of an echocardiography-based optimization approach yet also acknowledge some disadvantages. Echocardiography is time-consuming and not all patients have the time allowance to obtain echocardiographic images. Moreover, the inter- and intraobserver variability should be considered. Therefore, we adjusted device parameters, such as the VV and AV intervals at rest. This might be suboptimal since there are studies that demonstrate variability in these parameters at rest and during exercise [26, 27]. Nevertheless, we were able to show that there was a significant improvement in LVEF and NYHA classes after adjusting these settings at rest.

**Fig. 5.**
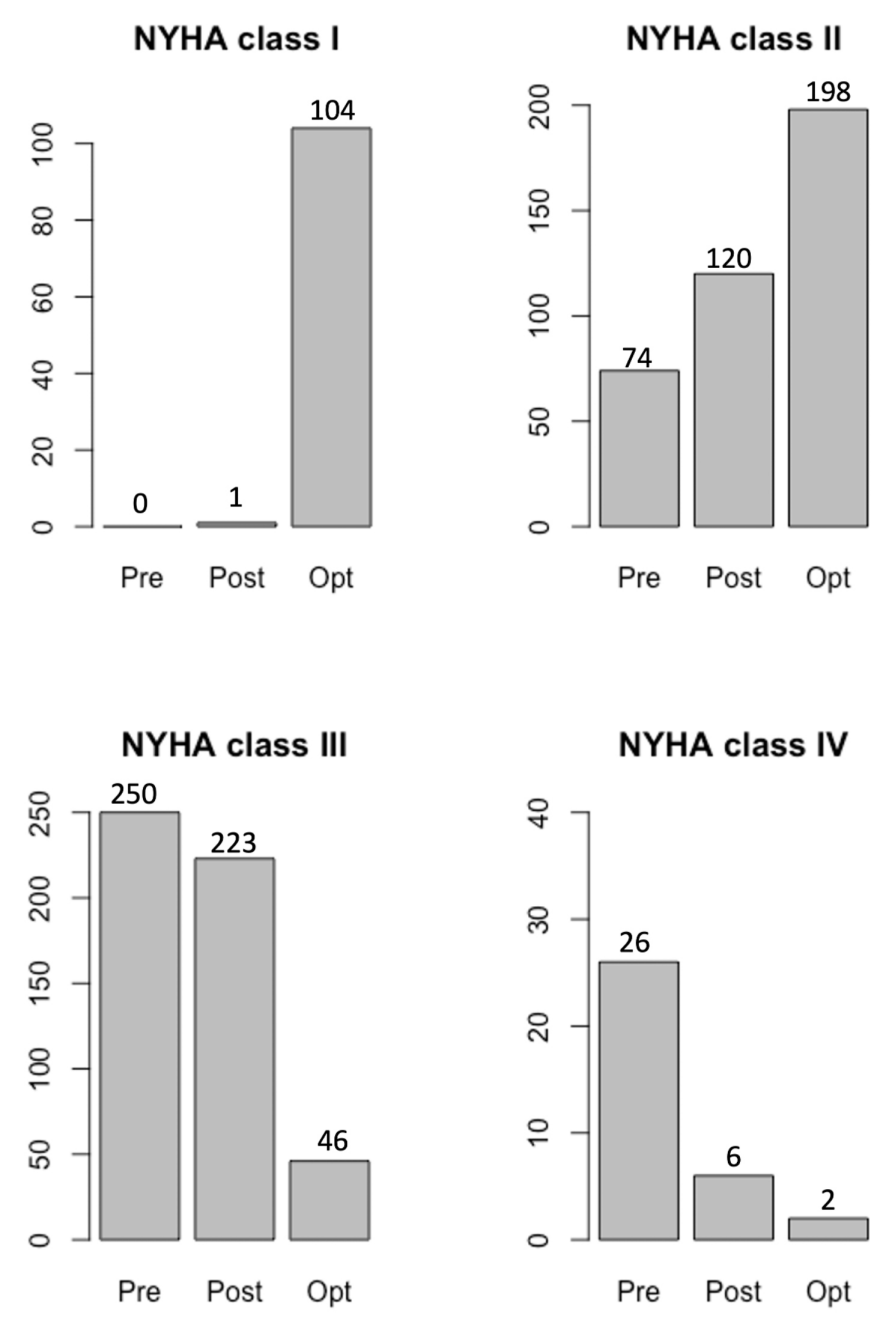
**Improvements in NYHA class after CRT optimization. **Abbreviations: Pre, before CRT implantation; Post, after CRT implantation but before CRT optimization; Opt, after CRT optimization; NYHA, New York Heart Association classification; CRT, cardiac resynchronization therapy. After CRT implantation, the NYHA class improved in all 350 patients, while the NYHA class further improved after optimization, with a greater number of patients exhibiting NYHA class I (104 patients). After optimization, 198 patients were in NYHA class II. The number of patients in NYHA classes III and IV were reduced to 46 and 2 patients, respectively, meaning the optimization resulted in a significant improvement *p* < 0.01.

In total, 67 patients exhibited an improvement in NYHA class after CRT implantation even before optimization was achieved (Fig. 5). We believe that performing any intervention could have a positive effect on the well-being of each patient. It is difficult to determine if this improvement was solely due to our CRT optimization since this study was not performed as a double-blind. In this study, we do not compare different methods of CRT optimization and we do not claim that other methods are less effective than our method. This study aimed to only detail our positive experiences from using the described method of CRT optimization to illustrate that CRT optimization remains effective.

## 5. Conclusions

CRT optimization remains valuable in improving LVEF and functional statuses measured using the NYHA class in all patients who have received CRT devices. In this study, we share our positive experience of using a multidisciplinary team to perform protocol-driven echocardiography-based CRT optimization.

## Data Availability

All data and materials are available upon request.
